# Supplementary material for: High Thermoelectric Performance of a Novel γ-PbSnX2 (X = S, Se, Te) Monolayer: Predicted Using First Principles
Source: Nanomaterials (Basel). 2023 Apr 29;13(9):1519. doi: 10.3390/nano13091519 (PMC10180089; doi:10.3390/nano13091519)
Supplement: Supplementary file 1 [file nanomaterials-13-01519-s001.zip › nanomaterials-2376453-supplementary.pdf]

nanomaterials

MDPI

Supplementary Materials

High Thermoelectric Performance of a Novel γ-PbSnX2 (X=S, Se, Te) Monolayer: Predicted by First-Principles

Changhao Ding, Zhifu Duan, Nannan Luo, Jiang Zeng, Wei Ren *, Liming Tang and Keqiu Chen *

Department of Applied Physics, School of Physics and Electronics, Hunan University, Changsha 410082, China

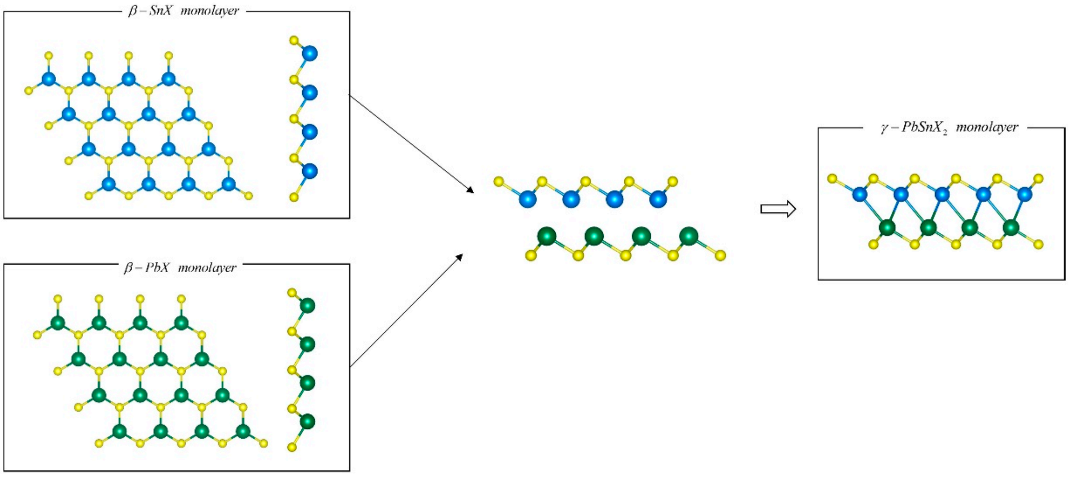

Figure S1. The structure of γ-PbSnX2(X=S, Se, Te) and its possible synthesis process.

Computation Method of Electronic and Phonon Transport

The electronic transport coefficients are evaluated by solving the semiclassical Boltzmann transport theory (BTE) with the relaxation time approximation (RTA), as performed in the BoltzTraP code. The electrical conductivity, Seebeck coefficient and electronic thermal conductivity are calculated by the formula as follows:

$$\sigma(\mu,\mathrm{T})=\mathrm{e}^2\int d\varepsilon(-\frac{\partial f_\mu(T,\varepsilon)}{\partial\varepsilon})\Xi(\varepsilon) \quad \text{(S1)}$$

$$S(\mu,\mathrm{T})=\frac{ek_B}{\sigma}\int d\varepsilon(-\frac{\partial f_\mu(T,\varepsilon)}{\partial\varepsilon})\Xi(\varepsilon)\frac{\varepsilon-\mu}{k_BT} \quad \text{(S2)}$$

$$\kappa_e=k_B{}^2T\int d\varepsilon(-\frac{\partial f_\mu(T,\varepsilon)}{\partial\varepsilon})\Xi(\varepsilon)(\frac{\varepsilon-\mu}{k_BT}) \quad \text{(3)}$$

where $f_\mu(T,\varepsilon)=\frac{1}{e^{(\varepsilon-\mu)/k_BT}+1}$ is the Fermi–Dirac distribution function, k_B is Boltzmann's constant, ε is the energy of carriers, and $\Xi(\varepsilon)=\sum_k v_k\otimes v_k\tau_k$ is the transport distribution function, in which v_k and τ_k are the group velocity and relaxation time at wave vector k. To obtain reliable electronic transport

Coefficients, a denser Monkhorst–Pack k-mesh with $60\times60\times1$ is used in the first Brillouin zone. The relaxation time of acoustic phonon is obtained by deformation potential (DP) theory based on effective mass approximation, which is determined by $\tau=\frac{\mu m^*}{e}$

, where μ and m^* are the carrier mobility and effective mass, respectively. The carrier mobility can be calculated by $\mu = \frac{e\hbar^3 C_{2D}}{k_B T m^* m_d E_1^2}$, where $C_{2D} = \frac{1}{S_0}\frac{\partial^2 E}{\partial(\Delta L/L_0)^2}$ is the elastic modulus and $E_1 = \frac{\partial E}{\partial(\Delta L/L_0)}$ is the DP constant. The effective mass can be calculated by $m^* = \frac{1}{\hbar^2}\frac{\partial^2 E(k)}{\partial k^2}$.

For the phonon calculations, the second-order harmonic interaction force constants (2nd IFCs) and phonon frequencies are calculated via density functional perturbation theory (DFPT). The 2nd IFCs are obtained using a 3 × 3 × 1 supercell with a 3 × 3 × 1 k-mesh, and the third-order anharmonic IFCs (3rd IFCs) are obtained using a 3 × 3× 1 supercell with the finite-difference method. The interactions with the sixth-nearest neighbor atoms are considered for 3rd IFCs. The phonon transport properties are evaluated by solving the phonon Boltzmann transport equation with second order harmonic and third order anharmonic interatomic force constants, as performed in the ShengBTE code. In order to generate an accurate lattice thermal conductivity, a denser 60 × 60 × 1 k mesh is adopted.

Table S1. The energy of γ-$PbSnX_2$ monolayer and a single atom (Pb, Sn and X=S, Se, Te) of structure, and the formation enthalpy (ΔH) of γ-$PbSnX_2$ monolayer.

Material	H_{PbSnX2}(eV)	H_{Pb}(eV)	H_{Sn}(eV)	H_S(eV)	H_{Se}(eV)	H_{Te}(eV)	ΔH(eV/atom)
γ-$PbSnS_2$	−17.45			−4.13			−0.45
γ-$PbSnSe_2$	−15.87	−3.55	−3.84		−3.5		−0.37
γ-$PbSnTe_2$	−14.60					−3.14	−0.23

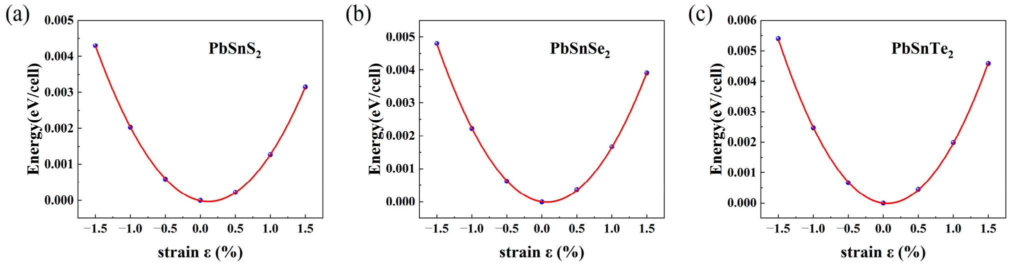

Figure S2. The energy-strain relationship of γ-PbSnX2(X = S, Se, Te).

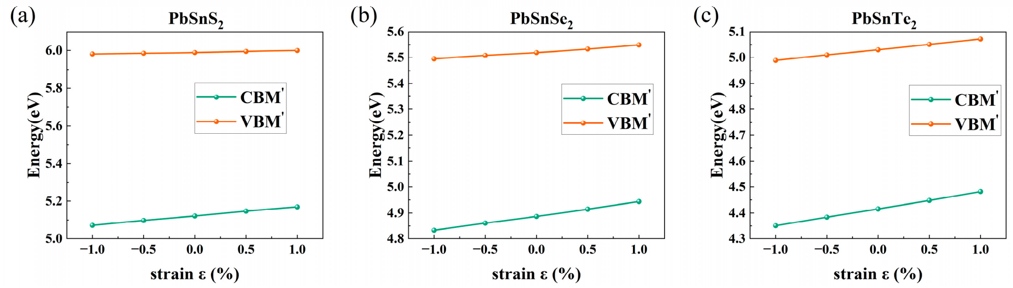

Figure S3. Calculated band energies of the CBM and VBM of γ-PbSnX2 (X = S, Se, Te) with respect to the vacuum energy as a function of uniaxial strains. The red solid lines are linear fitting curves. The fitted slopes are presented, which correspond to the DP.
